# Outcome of Dome Osteotomy With Plate Osteosynthesis for Genu Valgum in Late Adolescents and Young Adults

**DOI:** 10.7759/cureus.7894

**Published:** 2020-04-29

**Authors:** Kuldeep Bansal, Puneet Mishra, Manish Chadha, Pratyush Shahi, Rahul Anshuman, Aditya N Aggarwal

**Affiliations:** 1 Orthopaedics, University College of Medicine Sciences and Guru Teg Bahadur Hospital, Delhi, IND; 2 Orthopaedics, University College of Medical Sciences, Delhi, IND; 3 Orthopaedics, University College of Medical Sciences and Guru Teg Bahadur Hospital, Delhi, IND

**Keywords:** genu valgum, dome osteotomy, plate osteosynthesis, late adolescents, young adults, philos

## Abstract

Aim

To evaluate the degree of correction and outcomes after correction of genu valgum deformity using dome osteotomy with plate osteosynthesis in late adolescents and young adults.

Methods

A total of 27 knees in 21 patients underwent correction using dome osteotomy fixed with 3.5-mm low-profile proximal humeral locking system (PHILOS) plate. The functional, clinical, and radiological assessments were performed preoperatively and at six months postoperatively. Functional assessment was performed using the Bostman score, while clinical and radiological assessments were performed by measuring intermalleolar distance, tibiofemoral angle, mechanical lateral distal femoral angle, and mechanical axis deviation. All values were compared preoperatively and postoperatively using the paired t-test and Wilcoxon’s test.

Results

The comparison between preoperative and postoperative data was statistically significant (P<0.0001). Twenty patients had an excellent knee score, and one patient had a good score. None had an unsatisfactory score.

Conclusions

Dome osteotomy fixed with well-contoured, 3.5-mm low-profile PHILOS plate allows deformity correction at the CORA (center of rotation of angulation) of the knee and permits early knee mobilization without significant procedure or implant-related complications with excellent outcomes.

## Introduction

Genu valgum or knock-knees in adolescents and young adults is a frequent cause of orthopedic referral [1,2]. Coronal plane malalignments may be idiopathic or secondary to trauma or osteomalacia. These malalignments increase the risk of premature tibiofemoral arthritis due to eccentric single compartment loading and may lead to anterior knee pain, patella-femoral problems owing to increased Q-angle, circumduction gait, and difficulty in running.

Significant valgus deformity requires surgical intervention to improve the knee biomechanics. Therefore, surgical treatment may become necessary depending upon the degree of the deformity and age of the patient in order to prevent late-onset degeneration and arthritis and to improve performance in sports and related activities [3]. Malalignment can be unilateral or bilateral. When the physis is open, gradual growth modulation with stapling, percutaneous drill hemiepiphysiodesis, and transphyseal screws can correct the deformity. These methods cause permanent growth arrest; therefore, an exact calculation of remaining growth and perfect timing for the surgical procedure is required. Temporary epiphysiodesis is reversible and therefore can be performed at an earlier age; once the implant is removed, there is a resumption of the growth again and this can be done using eight-plate stapling [4,5].

If the valgus malalignment is significant in a skeletally mature patient, one can perform corrective osteotomy with or without internal or external fixation. The deformity may originate from the distal femur, proximal tibia, or knee joint. The most common cause of the deformity is in the distal femur. Based on the location of the deformity, genu valgum can be corrected either by femoral supracondylar or high tibial osteotomy. Various types of corrective osteotomies of the distal femur have been described in the literature, such as lateral opening wedge, medial closing wedge, dome osteotomy, wedge-less ‘V’ osteotomy, and Ilizarov’s distraction method, each with its own advantages and disadvantages [6,7].

Dome osteotomy is cylindrical shaped, and its bone cuts rotate around the central axis of the deformity. It provides high adjustability of the bone ends, good bony contact, and better stability. These advantages make it more effective than transverse osteotomy for deformity correction, particularly around the knee joint [8]. As no bone is resected, it avoids limb length discrepancy. It corrects the deformity without producing a translation at the osteotomy site [9].

The literature on the functional outcome of dome osteotomy with plate osteosynthesis is scarce, and the majority of studies have been conducted in the West. As India is a developing country and as nutritional deficiencies manifesting as genu valgum are very common, we prospectively envisaged studying the clinical, radiological, and functional outcomes after correction of genu valgum deformity of the distal femoral origin using femoral supracondylar dome osteotomy with internal fixation using low-profile proximal humeral locking plate in late adolescents and young adults.

## Materials and methods

A total of 21 patients (27 knees), comprising adolescents and young adults, with symptomatic genu valgum deformity originating from the distal femur and associated with gait disturbance, difficulty in running, knee discomfort, patellar malalignment, or cosmetic concern were enrolled in the study after obtaining informed consent. Only cases with bilateral genu valgum deformity with intermalleolar distance (IMD) more than 10 cm, unilateral genu valgum deformity with tibiofemoral angle more than 15 degrees, mechanical axis deviation (MAD), mechanical lateral distal femoral angle (mLDFA) less than 84 degrees with normal medial proximal tibial angle (MPTA), fused distal femoral physis on X-ray examination, and minimum postoperative follow-up of six months were included.

A thorough history of onset and progression was sought in all patients. The deformity assessment was performed clinically and radiologically. Clinically, the knee flexion test was used to assess the origin of the deformity [10]. Clinical tibiofemoral angles and IMDs were noted in all patients initially and at a minimum of six months or at the latest follow-up. In the standing position with the patella facing forward, anterior superior iliac spine (ASIS), the center of the patella, and the center of the ankle (point midway between the medial and lateral malleoli) were marked. ASIS and the center of the patella were joined, and the center of the patella and the center of the ankle were joined by two lines. Using a goniometer, the tibiofemoral angle was measured using the above lines [11]. The IMD was measured in the standing position with the patella facing forward and the knees in extension and just touching each other.

The functional assessment was performed using the Bostman et al. knee score [12]. Patients with a score between 28 and 30 were classified as having an excellent outcome. A score between 20 and 27 was classified as a good outcome, and a score below 20 was classified as an unsatisfactory outcome.

**Figure 1:**
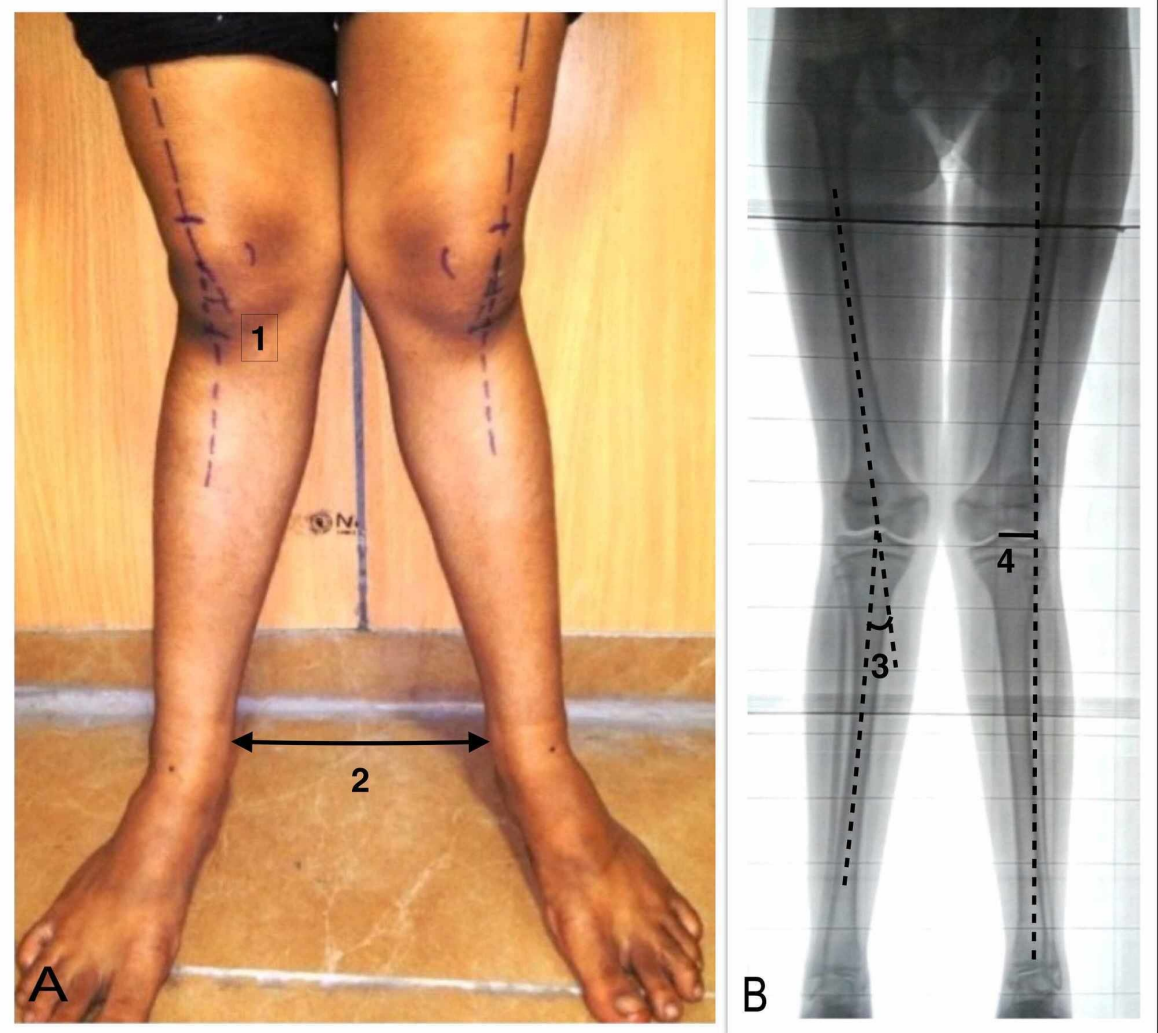
Preoperative clinical and radiographic assessment. (A) Measurement of theclinical tibiofemoral angle (1) and IMD (2). (B) Measurement of the radiological tibiofemoral angle (3) and mechanical axis deviation (4).

Radiologically, all enrolled patients were subjected to the standing anteroposterior (AP) long limb films (X-ray scanogram), in which both lower limbs were radiographed together from the hip, knee, and ankle joints, and the standard weight-bearing X-ray (AP and lateral) views of the involved knees. The radiological tibiofemoral angle (angle formed between the anatomical axes of the tibia and the femur) was measured and noted in all patients initially and at a minimum of six months or at the latest follow-up. The mechanical axis of the lower limb was drawn by joining the line from the center of the femoral head to the center of the ankle joints. The distance between the center of the knee joint and the mechanical axis was measured as MAD. The mLDFA was calculated between the mechanical axis of the femur and the articular surface of the distal femur. The MPTA was measured between the tibial mechanical axis and the articular surface of the proximal tibia (Figure 1) [13].

The blood investigations including serum calcium/phosphate, serum alkaline phosphatase, and kidney function tests were conducted. Patients with active underlying metabolic disorders such as rickets and osteomalacia were first provided with medical management prior to surgery.

All the patients were operated under appropriate anesthesia (general/spinal/epidural) in the supine position on a radiolucent operating table for C-arm guidance and with a pneumatic tourniquet applied to the proximal thigh. The involved knee was flexed to 60 degrees during the surgery to avoid pressure in the popliteal area by keeping a large bolster under it. During draping, care was taken to expose the ankle so that the center of the ankle could be determined easily. An ECG (electrocardiogram) electrode was applied on the skin overlying the center of the femoral head, and another electrode was applied on the skin overlying the center of the ankle joint. A second generation cephalosporin was given at induction 20 minutes before tourniquet inflation.

**Figure 2:**
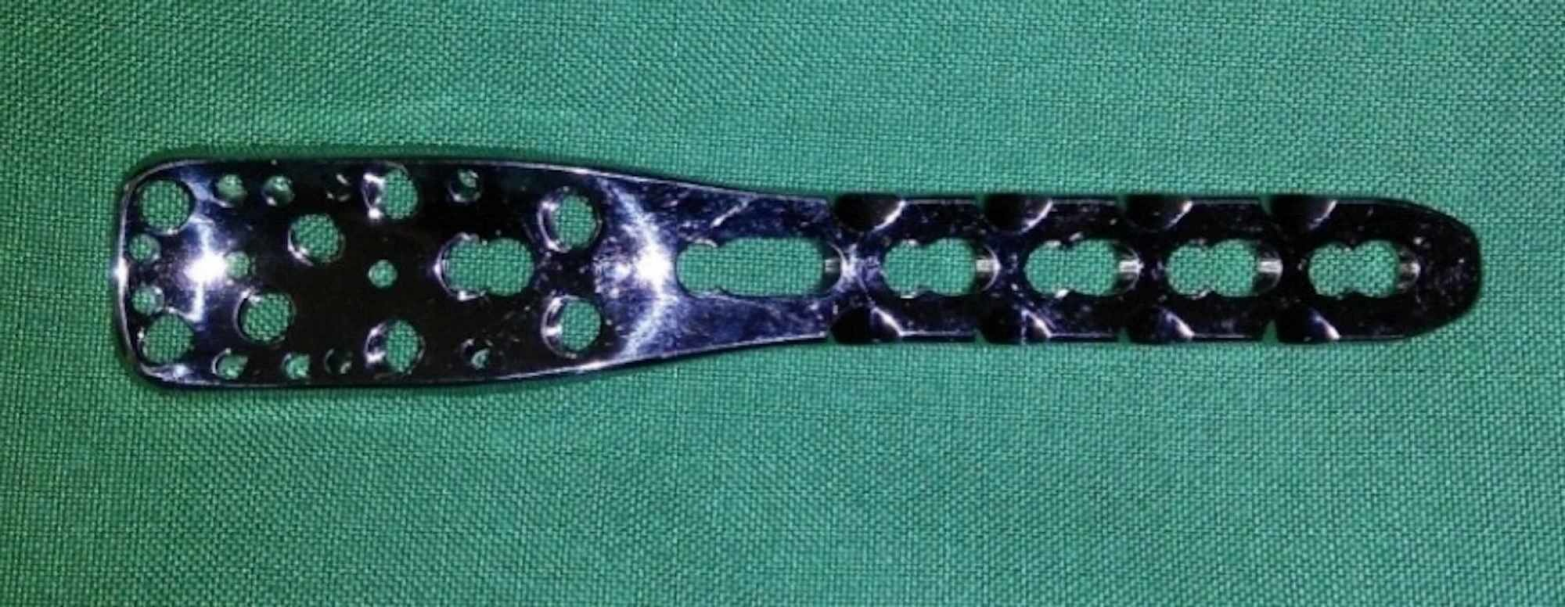
A 3.5-mm low-profile proximal humeral locking plate

A 10-cm lateral incision was made in the distal thigh along the midline of the femoral shaft extending up to the lateral knee joint line. Using the posterolateral approach, the vastus lateralis was elevated from the lateral intermuscular septum. The lateral and anterior femoral cortices in the supracondylar area of the distal femur were exposed. A blunt-tipped Hohmann retractor was then placed medially exposing the anteromedial cortex, and a second wide retractor was placed subperiosteally posterior to the distal femur to protect the vascular bundle. At this time, a 3.5-mm proximal humerus locking plate (Figure 2) with appropriately numbered holes was provisionally placed at the lateral flare of the lateral distal femoral condyle, and a dome-shaped circular osteotomy line with its convexity superior was marked on the distal femur in the metaphyseal area such that future plate placement for osteotomy fixation allowed placement of at least four distal locking screws in the distal femur. Then, at the selected site (metaphyseal area, approximately fingerbreadth above the medial femoral condyle), a circular line with its convexity superior was drawn using electrocautery. A 2.5-mm drill bit and drill sleeve were used to create drill holes along the circular line, just penetrating the posterior cortex. The holes were created close to each other to ensure a smooth circular shape.

A 1/4th-inch osteotome was then used to connect the drill holes, except at the corners, where a curved gauge or osteotome was used to complete the osteotomy. Then carefully a 1/4th-inch osteotome was used to complete the osteotomy medially, taking care not to advance the osteotome too medially or too posteriorly, and the osteotomy was then completed slowly by osteoclasis.

The leg was then manipulated into valgus until the knee joint line became horizontal and the deformity was clinically corrected. The corrected deformity was then provisionally fixed with two crossed Kirschner wires (K-)wires (2 mm) each passing medially and laterally from either femoral condyles into the supracondylar area. Deformity correction and overall limb alignment were then checked by an electrocautery cord that was used from the femoral head marker to the mid-ankle marker, and the knee was viewed under C-arm to ensure that this line was corrected to the center of the knee or just lateral to the medial tibial spine. The osteotomy fixation was then completed using a 3.5-mm proximal humerus locking plate in dual mode (compression and locking mode) using at least four distal locking screws and four to five proximal screws in the supracondylar distal femur, ensuring at all times that the distal femoral correction achieved was well maintained during application of plate also. Tourniquet was released, and following hemostasis control, the incision was closed in layers over a drain, and a postoperative fixed knee brace was applied. Tourniquet time was noted.

The drain was removed at 48 hours postoperatively, and drain output was noted in all patients. The surgical site was inspected and dressed, and postoperative radiographs of thigh including the knee (AP and lateral views) were taken. Gentle passive and active-assisted knee range of mobilization and isometric quadriceps exercises were started on the second postoperative day and gradually progressed as tolerated by the patient under adequate oral analgesia control. Non-weight bearing walking was commenced on the second postoperative day and was continued until initial healing of the osteotomy was confirmed radiologically, usually after six weeks of follow-up. During this time, knee mobilization and exercises for building muscle strength were recommended to the patient. Patients were kept on a regular follow-up at two weeks, six weeks, three months, six months, and at the latest follow-up. Postoperative X-rays of the operated thigh with the knee (AP and lateral views) were again taken at six weeks and at three months to assess the healing of the osteotomy.

**Figure 3:**
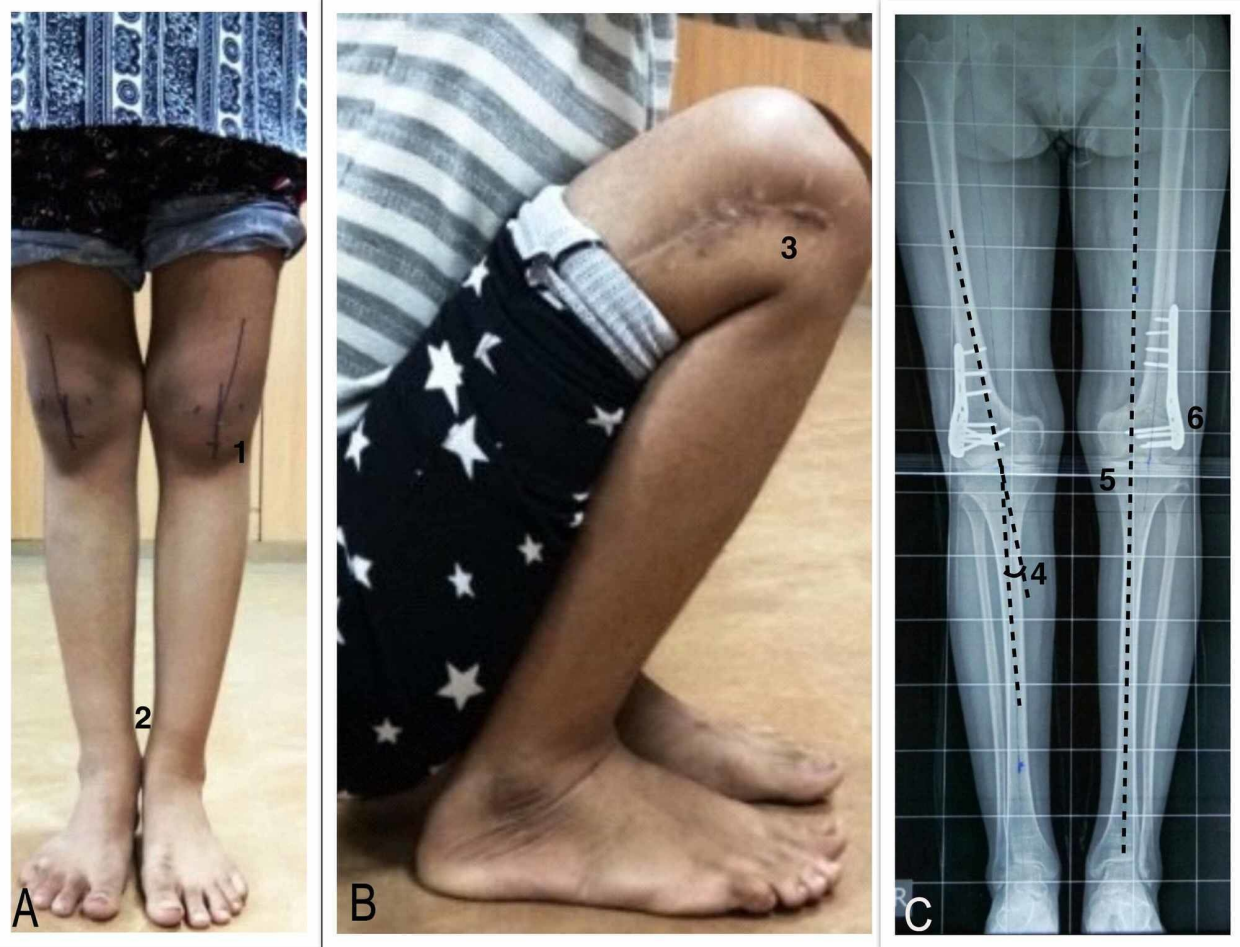
Postoperative clinical and radiographic assessment. A) Measurement of the clinical tibiofemoral angle (1) and the medial malleoli touching each other after the correction of bilateral deformities (2). (B) Full flexion at both knee joints with (3) well-healed surgical scar. (C) Measurement of the radiological tibiofemoral angle (4), mechanical axis passing medial to the tibial spine (5), and 3.5-mm low-profile proximal humeral locking plate (6).

A repeat X-ray scanogram of the patient was performed at six months. Radiological parameters such as MAD, mLDFA, radiological tibiofemoral angles, clinical tibiofemoral angle, and IMDs were measured to assess the degree of deformity correction. Functional outcome scoring using the Bostman et al. knee scoring was performed at six months and/or at the latest follow-up (Figure 3).

Categorical variables were presented as number and percentage, and continuous variables were presented as mean ± standard deviation and median. The normality of the data was tested using the Kolmogorov-Smirnov test. If the normality was rejected, then a non-parametric test was used. Quantitative variables were compared using the paired t-test/Wilcoxon’s test across follow-ups. Quantitative variables were compared using the independent t-test/Mann-Whitney test between two groups. Qualitative variables were correlated using the chi-square test. A P value of <0.05 was considered statistically significant. The data were entered in Microsoft Excel spreadsheet (Microsoft Corp., Redmond, WA, USA), and analysis was performed using SPSS Version 20.0 (IBM Corp., Armonk, NY, USA).

## Results

The mean age of the patients was 17.1 years. Out of the 21 patients, 13 were females and 8 were males; 3 patients had a unilateral deformity, whereas 18 had bilateral deformities, out of which 12 opted for unilateral deformity correction. One limb was operated at a time in bilateral cases.

IMD and the clinical tibiofemoral angle were included in the clinical criteria. The mean IMD before surgery was 14.5 cm, which improved to 6.8 cm after surgery. The difference was statistically significant (P < 0.0001). The mean clinical tibiofemoral angle before surgery was 20.4 degrees in bilateral cases and 18.6 degrees in unilateral cases, which improved to 12.9 and 8.6 degrees, respectively, after surgery. Both the differences were statistically significant (Table 1). The mean tourniquet time was 65.6 minutes, and the mean drain output at first dressing was 222.2 mL.

**Table 1:** Intermalleolar distance and clinical tibiofemoral angle.

Measurement	Mean preoperative	Mean postoperative	P-value
Intermalleolar distance (cm)	14.52±5.19	6.85±3.47	<0.0001
Clinical tibiofemoral angle (degree)	Bilateral: 20.35±4.9	Bilateral: 12.92±3.6	<0.0001
	Unilateral: 18.67±67	Unilateral: 8.67±1.15	<0.004

Radiological tibiofemoral angle, MAD, mLDFAs, and MPTAs were included in the radiological criteria. The preoperative mean radiological tibiofemoral angle was 13.0 degrees in bilateral cases and 14.7 degrees in unilateral cases, which improved to a postoperative mean of 8.9 degrees and 7.1 degrees, respectively. The preoperative mean MAD was 11.7 mm, which improved postoperatively to 5.5 mm. The preoperative mean mLDFA was 74.6 degrees, which improved to 81.6 degrees postoperatively. All these differences were statistically significant (Table 2). The mean MPTA was 86.2 degrees (range: 84-89 degrees).

**Table 2:** Radiological tibiofemoral angle, MAD, and mLDFA. mLDFA, mechanical lateral distal femoral angle; MAD, mechanical axis deviation

Measurement	Preoperative mean	Postoperative mean	P-value
Radiological tibiofemoral angle (degree)	Bilateral: 13.04±2.82	Bilateral: 8.9±1.89	0.0001
	Unilateral: 14.67±0.58	Unilateral: 7.17±0.76	0.002
mLDFA (degree)	74.59±5.2	81.57±4.01	0.0001
MAD (mm)	11.72±3.64	5.54±2.97	0.0001

The functional scoring was performed using the Bostman et al. score. The mean preoperative score was 28.7, which improved to 29.6 at six-month follow-up; this was statistically significant. Out of 21 patients, 20 had an excellent postoperative knee score of more than 29 points and 1 patient had a knee score of 21 points graded as good. None of the patients had an unsatisfactory knee score.

Among complications, there was one case of superficial wound infection and one case of loss of correction of the deformity. Three patients developed hypertrophic scars at the surgical site. Union at osteotomy site was achieved in all the patients approximately around six to eight weeks postoperatively, which was defined by bridging callus in at least three out of four cortices across the osteotomy site in AP and lateral views. None of the patients had implant impingement or knee stiffness.

## Discussion

The leading causes of genu valgum deformity in the developing and developed countries are nutritional rickets and trauma, respectively. Persistent genu valgum leads to abnormal gait, functional disturbances, premature arthritis, anterior knee pain, patellar maltracking leading to patella-femoral subluxation, and difficulty in running. The aim of treatment is to correct the limb alignment and hence prevent subsequent joint degeneration. For correction of valgus alignment in late adolescents and young adults, various distal femoral osteotomies have been described in the literature. Since physes are already fused, growth modulation is not a reliable modality of treatment in these patients. In our study, we evaluated the functional outcome of distal femoral dome osteotomy fixed with a 3.5-mm low-profile proximal humerus locking plate in late adolescents and young adults with genu valgum deformity of distal femoral origin.

In clinical assessment, both IMD and clinical tibiofemoral angle showed significant improvement after surgery. A similar result was noted in other studies [14-16]. Functional assessment using the Boston et al. score also showed significant improvement, similar to the findings of Gupta et al. and those of Agarwal and Shaharyar using the same score [14,17]. In radiological assessment, radiological tibiofemoral angle, MAD, and mLDFA all showed significant improvement, a finding that was in accordance with various other studies [14,16,18,19].

The lateral approach was used, and the quadriceps muscle was retracted from the intermuscular septum, thus avoiding the probable damage to the quadriceps and its subsequent tethering at the osteotomy site. Due to rigid internal fixation and early postoperative knee mobilization, further tethering and damage to the quadriceps were prevented, as previously noticed by Gautam et al. [16]. The osteotomy was created using multiple drill holes, which provided additional stability by producing small bone spikes interdigitating at the osteotomy site. Dome osteotomy was preferred due to the high adjustability of the bone ends, good bony contact, and better stability, with no limb length discrepancy. Watanabe et al. stated that a difference in the centers of the deformity and osteotomy in dome osteotomy led to an imprecise correction [9]. In contrast to this, our study showed that executing a dome osteotomy with the CORA (center of rotation of angulation) at the knee and realigning the mechanical axis to the center of the knee can achieve deformity correction even when osteotomy was fixed internally. Various methods used to fix the osteotomy have been described in the literature. In our study, a well-contoured 3.5-mm low-profile proximal humeral locking plate in dual mode was used to fix the osteotomy, which helped in early rehabilitation and early weight-bearing. None of our cases reported any symptomatic implant irritation. This might be because of better plate contouring over the distal femoral condyle after osteotomy and low profile of the plate. Locking plates lead to mechanical rigidity at the osteotomy site, However, they have the associated disadvantages such as hardware failure in distraction mode, painful hardware, failure to correct the residual deformity after surgery, and risk of bone loss under the plate [20]. Kazemi et al. reported four (20%) cases of nonunion in the locking compression plate group [19]. In contrast, despite our implant being a locking compression plate, there was no case of nonunion. This may be due to better plate contouring against the distal femoral lateral condyle and application of the plate in dual mode along with dome osteotomy causing maximum metaphyseal bony contact leading to optimal healing. Özcan et al. studied intramedullary nail versus plating for distal femoral osteotomy fixation and found that a retrograde intramedullary nail had better functional results as compared to a plate [21]. This was because of the irritating effect of the wide profile plate that led to a decrease in the knee range of motion of the patients treated with plating. In contrast, we did not observe any such decrease in the knee range of motion in any patient in our study. This might be because of the low-profile nature of the plate.

Gupta et al. reported two cases of a deep wound infection that subsided after implant removal [14]. In contrast, there was one case of superficial wound infection in our study that was diagnosed by redness, marginal necrosis, wound gap, and serous discharge with unhealthy granulation tissue, and responded well to one week of oral antibiotics.

Agarwal and Shaharyar reported one case of deep wound infection and one case of partial slippage of the lower femoral physis [17]. This slip was probably due to excessive force applied to achieve correction intraoperatively. On the contrary, we did not observe any such slip because physeal fusion was ensured before surgery in our study.

Watanabe et al. reported three cases of pin tract infections, three cases of delayed unions, one case of joint contracture, and one case of transient peroneal nerve palsy, but function recovered completely without treatment [9]. On the contrary, we did not encounter any peroneal nerve palsy even in severe deformity correction.

Prakash et al. fixed the osteotomy either by plaster or by K-wire if stability was doubtful and found five cases of superficial wound infections (all were patients in whom K-wires were used), three cases of limb-length discrepancy, two cases had a loss of reduction in the plaster, and one case of nonunion [15]. In our study, we had one case of loss of correction. This was due to a significant fall two months after the procedure, which led to plate and screw breakage. Plate and screw breakage was not seen in any other studies [14,15,18,19,22-24]. In our case, this might have been due to the significant trauma and due to lack of contour between the plate and osteotomy site, which might have led to increased tension on the implant-bone construct, thereby making the implant prone to breakage.

Gautam et al. reported hypertrophic scars at the surgical site in four knees, which was in accordance with our study as we reported hypertrophic scars in three knees [16].

We noted a female preponderance in patients in our study. This has also been reported by various other Indian studies [14-17]. Female patients and their parents were usually more concerned about the personal appearance and cosmesis due to deformity, which prompted them to seek surgical correction.

## Conclusions

Correction of symptomatic genu valgum deformity of distal femoral origin with supracondylar dome osteotomy and a 3.5-mm low-profile proximal humeral locking plate yield excellent clinical, radiological, and functional outcomes. However, future studies with more cases and longer follow-up comparing various low-profile plates with other types of osteotomies are needed.
